# Targeting Human Osteoarthritic Chondrocytes with Ligand Directed Bacteriophage-Based Particles

**DOI:** 10.3390/v13122343

**Published:** 2021-11-23

**Authors:** Aitthiphon Chongchai, Sajee Waramit, Tunchanok Wongwichai, Jirawan Kampangtip, Thanyaluck Phitak, Prachya Kongtawelert, Amin Hajitou, Keittisak Suwan, Peraphan Pothacharoen

**Affiliations:** 1 Thailand Excellence Centre for Tissue Engineering and Stem Cells, Department of Biochemistry, Faculty of Medicine, Chiang Mai University, Chiang Mai 50200, Thailand; itipolmk@gmail.com (A.C.); tunchanok.wong@gmail.com (T.W.); kpt.jirawan@gmail.com (J.K.); tphitak_mt@hotmail.com (T.P.); prachya.kongtawelert@gmail.com (P.K.); 2 Cancer Phagotherapy Group, Department of Brain Sciences, Burlington Danes Building, Hammersmith Hospital Campus, Imperial College London, London W12 0NN, UK; s.waramit15@imperial.ac.uk (S.W.); a.hajitou@imperial.ac.uk (A.H.)

**Keywords:** phage-based particle, targeted gene delivery, chondrocyte-affinity peptide, osteoarthritis

## Abstract

Osteoarthritis (OA) is a degenerative joint disease characterized by progressive deterioration and loss of articular cartilage. There is currently no treatment to reverse the onset of OA. Thus, we developed a targeted delivery strategy to transfer genes into primary human chondrocytes as a proof-of-concept study. We displayed a chondrocyte-affinity peptide (CAP) on the pIII minor coat protein of the M13 filamentous bacteriophage (phage)-based particle carrying a mammalian transgene cassette under cytomegalovirus CMV promoter and inverted terminal repeats (ITRs) cis elements of adeno-associated virus serotype 2 (AAV-2). Primary human articular chondrocytes (HACs) were used as an in vitro model, and the selectivity and binding properties of the CAP ligand in relation to the pathogenic conditions of HACs were characterized. We found that the CAP ligand is highly selective toward pathogenic HACs. Furthermore, the stability, cytotoxicity, and gene delivery efficacy of the CAP-displaying phage (CAP.Phage) were evaluated. We found that the phage particle is stable under a wide range of temperatures and pH values, while showing no cytotoxicity to HACs. Importantly, the CAP.Phage particle, carrying a secreted luciferase (*Lucia*) reporter gene, efficiently and selectively delivered transgene expression to HACs. In summary, it was found that the CAP ligand preferably binds to pathogenic chondrocytes, and the CAP.Phage particle successfully targets and delivers transgene to HACs.

## 1. Introduction

Advances in regenerative medicine technology significantly extend human longevity. Over the last decade, life expectancy has rapidly been extended, resulting in greater numbers of old-age population [1]. Osteoarthritis (OA) is a long-term chronic disease caused by the deterioration of joint cartilage, resulting in pain, impaired movement, and disability. The disease is the most common cause of disability in older adults. The United Nations speculated that 40 million people aged over 60 will be severely disabled because of OA by 2050 [2]. Treatment of OA is emphasized in palliative medical approaches, including pain management and improving quality of life; however, there is no treatment that ameliorates or reverses the onset of OA [3]. There were attempts to target and deliver therapeutic genes to reverse pathogenic chondrocytes back to normal using non-viral or viral vectors as carriers [4]. The disadvantages of the non-viral vectors include toxicity and instability [5], while the viral vectors have been widely employed as promising tools for gene therapy. Several gene therapies in clinical trials in humans were mediated by viral vectors, including lentivirus, adenovirus, and adeno-associated virus (AAV) [6]. Recently, the AAV vector was utilized to deliver therapeutic genes to chondrocytes via the intra-articular delivery route [7]. The vector showed promising results and has entered clinical testing; however, AAV vectors are unstable under high temperatures, show non-specific cellular up-take, and are unsuitable for systemic administration. To overcome those limitations, the development of a non-toxic chondrocyte-targeting vector is an unmet need.

The chondrocyte-affinity peptide (CAP) was identified, and it was suggested that this ligand could specifically bind to chondrocytes [8]. Although its binding receptor has not yet been elucidated, the peptide is considered as an excellent candidate for chondrocyte targeting. Unfortunately, the CAP ligand itself cannot deliver therapeutic genes to the target site. A suitable nucleic acid carrier to combine with the CAP ligand is required. The ideal carrier should be non-toxic, suitable for non-invasive systemic administration, and able to accommodate large therapeutic genes. Adenoviral vectors can induce strong immune responses and cause multiple organ system failure, as reported in the death of an ornithine transcarbamylase-deficient patient who received adenoviral gene therapy [9,10]. Lentiviral or retroviral vectors can randomly incorporate into the host genome, causing oncogenic mutations. Leukemia was reported by an insertion of a retroviral vector adjacent to an oncogene promoter in an X-linked severe combined immunodeficiency patient who received retroviral gene therapy [11]. AAV is currently a preferable vector; however, its capsid has low transgene packaging capacity. Filamentous M13 bacteriophage (phage) has been developed into a drug delivery platform because of its potential advantages over mammalian viruses, including its safety profile, stability, low cost, and large packaging capacity for nucleic acid delivery. The phage genome was modified to accommodate mammalian transgene cassettes and ITRs elements from AAV2 to enhance gene transfer efficacy. The vector displaying a tumor homing peptide showed excellent tumor targeting efficacy after intravenous administration [12,13,14]. Therefore, we hypothesized that the combination of the CAP ligand with the phage vector can be a solution for the current limitations in chondrocyte-targeted gene delivery. Unlike mammalian vectors, phage vectors are prokaryotic viruses that infect bacterial cells only and lack native tropism for eukaryotic cells in general. Their coat proteins can be modified to display targeting molecules with minimal effect on the capsid structure. Systemic administration of the phages was proven safe and is widely used to treat antibiotic-resistant bacterial infections. In addition, the phages were approved for use as antibacterial additives in food production and processing [15], confirming their safety for other applications.

In this proof-of-concept study, we investigated the affinity of the CAP ligand on primary human chondrocytes isolated from articular cartilage. The CAP ligand showed good affinity towards osteoarthritic or inflamed primary human chondrocytes. We further constructed a chondrocyte-targeting phage vector by displaying the CAP ligand on the pIII minor coat proteins of phage capsids. The phage vector also displayed a histidine rich endosomal escape peptide, H5WYG, on recombinant pVIII major coat proteins to enhance gene delivery, as previously reported [14]. The modified phage particle showed stability within a wide range of pH and temperatures without causing toxicity to chondrocytes. We also proved that the M13 phage-based particle equipped with the CAP ligand can efficiently and selectively deliver transgene expression to chondrocytes.

## 2. Materials and Methods

### 2.1. Cell Culture

Primary human articular chondrocytes (HACs) from donors of various ages, as shown in Supplementary Table S2, and primary human synovial fibroblasts (PHSFs) were isolated from patients’ joints at Maharaj Nakorn Chiang Mai Hospital, Chiang Mai University, Chiangmai, Thailand with informed consent and local ethical committee approval (Ethic approval no. ORT-11-09-16A-14). We obtained samples from patients with osteosarcoma or chondrosarcoma with leg amputation. Since osteosarcoma or chondrosarcoma were mostly located at the specific sites above the knee, normal chondrocytes were isolated from knee and foot (no cancerous lesion). To minimize the possibility of having cancerous cell contamination, an orthopedic surgeon examined the cartilage prior to chondrocyte isolation. HACs were isolated from cartilage by dicing and digesting with collagenase type II (Sigma, Gillingham, Dorset, UK) in Dulbecco’s modified Eagle’s medium (DMEM, Sigma, Gillingham, Dorset, UK) at 37 °C for 24 h, then washing with Phosphate Buffer Saline (PBS). PHSFs were isolated by dicing the synovium and washing with PBS. The isolated HACs and PHSFs were cultured in DMEM (Sigma, Gillingham, Dorset, UK) supplemented with 10% Fetal Bovine Serum (FBS, Sigma, Gillingham, Dorset, UK), Penicillin (100 units/mL, Sigma, Gillingham, Dorset, UK), Streptomycin (100 μg/mL, Sigma, Gillingham, Dorset, UK), and L-glutamine (2 mM, Sigma, Gillingham, Dorset, UK). HACs were used for up to four passages. PHSF cells were used for three to six passages. Human chondrosarcoma cell line (SW1353) and human synovial sarcoma cell line (SW982) were purchased from American Type Culture Collection (ATCC). SW1353 cells were cultured in DMEM (Sigma, Gillingham, Dorset, UK) supplemented with 10% FBS (Sigma, Gillingham, Dorset, UK), penicillin (100 units/mL, Sigma, Gillingham, Dorset, UK), streptomycin (100 μg/mL, Sigma, Gillingham, Dorset, UK), and L-glutamine (2 mM, Sigma, Gillingham, Dorset, UK). SW982 cells were cultured in Leibovitz’s L-15 Medium (Sigma, Gillingham, Dorset, UK) supplemented with 10% FBS (Sigma, Gillingham, Dorset, UK), penicillin (100 units/mL, Sigma, Gillingham, Dorset, UK), streptomycin (100 μg/mL, Sigma, Gillingham, Dorset, UK), and L-glutamine (2 mM, Sigma, Gillingham, Dorset, UK). All the cells were maintained in a humidified atmosphere at 37 °C with 5% CO_2_.

### 2.2. Determination of CAP Ligand Binding Affinity to HACs versus PHSFs

HACs or PHSFs (5 × 10^6^ cells/mL) were resuspended in 0.05% (*w*/*v*) Bovine Serum albumin (BSA) in PBS. The cells were then incubated with FITC-labelled chondrocyte-affinity peptides (CAP; DWRVIIPPRPSA) or randomly scrambled peptide (SP; ARDWPIRPVPIS) with various concentrations from 1 to 8 μM in growing media for 4.5 h at 37 °C. The cells without the addition of the peptides were used as controls. After incubation, the cells were washed three times with PBS, then were centrifuged at 1000× *g* for 5 min. The cell pellets were resuspended in FACS buffer (PBS supplemented with 1%FBS) and subjected to flow cytometry (BD FACSAria™ III, Franklin Lakes, NJ, USA). For intracellular FITC-CAP visualization, HAC or PHSF monolayer cells, at 90% confluence, on 3.5 mm culture dishes were incubated with 4 μM of FITC-labelled CAP in growing media for 4.5 h at 37 °C. The cells were then washed 3 times with PBS. The cell nuclei were counter-stained and mounted with ProLong™ Gold Antifade Mountant with DAPI (Thermo Fisher Scientific, Waltham, MA, USA). The fluorescence-stained cells were observed under a fluorescence microscope (Zeiss Axio Scope.A1 Biology Microscope, Oberkochen, Germany).

### 2.3. IL-1β Treatment

HACs were trypsinized, counted using a haemocytometer, and seeded into 6-well plates. The cells were grown in a humidified atmosphere at 37 °C with 5% CO_2_ to reach 60–70% confluence. Prior to IL-1β treatment, the cells were cultured in serum-free media for 24 h, then treated with human IL-1β at 1, 2, and 10 ng/mL in growing media for 24 h.

### 2.4. Molecular Modelling 

CAP and SP sequences were retrieved from Pi Y. et al. (2011) [8]. Initially, the 3D models of each peptide were constructed using the Build-structure module in Discovery studio visualizer (BIOVIA, DS, 2019) [16]. These peptide sequences were further optimized for geometry by means of the steepest descents and conjugate gradients algorithms using the Forcite module within the Materials Studio 5.5 program [17]. Convergence of the calculation was considered to be achieved when the gradient of the atomic force was less than the preset value of 0.005 kcal mol^−1^ Å^−1^, the maximum displacement was 5 × 10^−5^ Å, and the energy was less than 1 × 10^−4^ kcal mol^−1^. Finally, the optimized-peptide models were superimposed and their molecular electrostatic potential maps were computed as seen in blue-to-red color for positive and negative charge, respectively.

### 2.5. RNA Extraction and RT-qPCR

Cells were collected and RNA was extracted using an RNA extraction kit (PureLink™ RNA Mini Kit, Thermo Fisher Scientific, Loughborough, UK) following the manufacturer’s protocol. RNA concentrations were measured with Nanodrop (Thermo Fisher Scientific, Loughborough, UK). One microgram of total RNA was converted to cDNA using a High-Capacity cDNA Reverse Transcription Kit (Applied Biosystems, Thermo Fisher Scientific, Loughborough, UK) following the manufacturer’s protocol. qPCR was performed to quantify the matrix metalloproteinase (*MMP*) *MMP-1, MMP-3,* and *MMP-13*, collagen *COL2A1*, and aggrecan *ACAN* gene expression using Powerup SYBR Green Master Mix (Thermo Fisher Scientific, Loughborough, UK) under the condition according to SYBR Green Master Mix manufacturer’s protocol. Primers were purchased from Life Technologies, Loughborough, UK; the primer sequences are listed in Supplementary Table S1. The level of gene expression of the samples was normalized to that of *GADPH* (the house-keeping gene), calculated using the 2^−ΔΔCT^ method.

### 2.6. Hyaluronic Acid (HA) ELISA 

A competitive enzyme-linked immunosorbent assay (ELISA) was used to measure HA in culture media. Microtiter 96 well plates (Maxisorp Nunc, Waltham, MA, USA) were coated with 100 μL per well 100 μg/mL of umbilical cord HA (Sigma, St. Louis, MO, USA) in the coating buffer (0.1 M NaHCO_3_, pH 9.6 in water) and incubated at 4 °C overnight. The plates were then blocked with 150 μL per well of 1% (*w*/*v*) BSA in PBS for 1 hour at room temperature. During the blocking step, 75 μL of culture media or standard competitors (HA Healon^®^: range 19–10,000 ng/mL, in 6% (*w*/*v*) BSA in PBS) were mixed with an equal volume of biotinylated hyaluronan-binding proteins (b-HABPs) (Sigma, St. Louis, MO, USA) at 1:200 dilution in 50 mM Tris-HCl, pH 8.6, in 1.5 mL microcentrifuges tubes. Next, blocking agent was discarded from the plates, and 100 μL of the mixtures in microcentrifuge tubes were transferred to the plates and incubated for 1 hour at room temperature. The plates were then washed with 0.05% tween20 in PBS three times, and incubated with 100 μL per well of peroxidase-mouse monoclonal anti-biotin (Sigma, St. Louis, MO, USA), at 1:2000 dilution in PBS, for 1 hour at room temperature. Next, the plates were washed with 0.05% tween20 in PBS three times, and 100 μL per well of the peroxidase substrate, (Sigma, St. Louis, MO, USA) in PBS were added and incubated at 37  °C for 5–10 min to allow color development. The reaction was stopped by adding 50 μL per well of 4 M H_2_SO_4_ and the absorbance ratio at 492/690 nm was measured using a Thermo Scientific Multiskan EX plate reader. The HA level was calculated using a standard curve.

### 2.7. DMMB Assay for Sulfated Glycosaminoglycans (s-GAGs) Levels

The levels of s-GAGs released in the culture media were measured by dimethyl-methylene blue (DMMB) assay [18]. Standard chondroitin sulfate-C (ranging from 0 to 40 μg/mL) or the culture media were added into a 96 well plate (50 μL/well), then two hundred microliters of DMMB solution were added. The complex of s-GAGs and DMMB was measured by using a microplate reader at 520 nm. The s-GAGs amounts were determined using a standard curve and shown as % s-GAGs release.

### 2.8. Cell Viability Assay 

The 3-(4,5-dimethylthiazol-2-yl)-2,5-diphenyl-2H-tetrazolium bromide (MTT) assay was used to measure cell viability. Cell culture medium was replaced with 100 μL of MTT reagent (5 mg/mL in PBS) and incubated for 4 h at 37 °C, 5% CO_2_. After incubation, the MTT reagent was gently removed and DMSO was added to dissolve the complex. Finally, the absorbance was measured with a microplate reader using the absorbance ratio at 540/630 nm. Cell viability was interpreted by comparison to control untreated cells.

The Trypan Blue Exclusion test was also used to determine cell viability. The transduced cells were trypsinized and centrifuged at 1000× *g* for 5 min. The cells were resuspended in PBS and diluted by mixing with 0.4% trypan blue (1:1 dilution). The mixture was then incubated 3 min at room temperature and a drop of the trypan blue/cell mixture was applied to a haemacytometer, and then the unstained (viable) and stained (nonviable) cells were counted separately under a light microscope. The percentage of viable cells was calculated by comparison to control untreated cells.

### 2.9. Electron Microscope Imaging of Phage Particles

The formvar carbon-coated 200-mesh grids were glow discharged at 10 mA for 2 min. The solutions of bacteriophage particles were added on the grids then incubated for 1 min and removed by blotting on absorbent paper. Next, the grids were washed with deionized water and blotted on absorbent paper. The grids were then floated on a drop of deionized water for 3 min and blotted on absorbent paper. After that, the grids were floated on a drop of 1% glutaraldehyde for 5 min and blotted on absorbent paper. The grids were then negatively stained by applying 1% uranyl acetate to the grid for 4 min and blotted on absorbent paper. Finally, the grids were used to take images using a transmission electron microscope (JEOL JEM-2010, Tokyo, Japan) and analyzed using ImageJ software.

### 2.10. Construction and Production of CAP.Phage Particle

To generate bacteriophage-derived particles for targeted gene delivery to chondrocytes, the M13KO7 (NEB, Hitchin, Hertfordshire, UK) plasmid was genetically manipulated to display copies of CAP (DWRVIIPPRPSA) on the pIII minor coat proteins. Primer pairs that specifically bind to the pIII region of M13KO7 plasmid were designed (Table S1). The primers were flanked with CAP and GGGGS linker DNA sequence. PCR reaction was set up using Q5 DNA polymerase (NEB, Hitchin, Hertfordshire, UK) and performed according to the manufacturer’s protocol. The recombinant CAP-M13KO7 product was circularized with T4 DNA ligase (NEB, Hitchin, Hertfordshire, UK) and transformed into *E. Coli* DH10B for amplification and positive clone screening. The positive recombinant CAP-M13KO7 clones were verified for correction and alignment with DNA sequencing (Eurofins DNA sequencing service, Ebersberg bei München, Germany). The histidine-rich endosome escape peptide H5WYG (GLFHAIAHFIHGGWHGLIHGWYG) was displayed on the recombinant pVIII proteins as described in Suwan K. et al. (2018) [14]. The particle also carried a mammalian gene expression cassette flanked by ITR cis elements from AAV2. The gene expression cassette encoded a cytomegalovirus (CMV) promoter-driven secreted luciferase (*Lucia*) reporter gene. To construct phage-based vector-expressing plasmid-containing *Lucia* gene, the *GFP* gene from the pAAV-*GFP* control plasmid (Cell Biolabs, Cambridge, UK) was replaced with the *Lucia* gene (InvivoGen, Toulouse, France) under the control of a CMV protomer.

The untargeted phage (M13.Phage) displaying only the H5WYG peptide on recombinant pVIII without pIII minor coat protein modification was included as a control vector. The bacteriophage-based particle was produced using host bacteria (*Escherichia coli TG1*). Briefly, the plasmid of the phage-based vector was transformed into the *E. Coli* TG1 strain (Zymo Research, Irvine, CA, USA) according to the manufacture’s protocol. The bacteria culture was grown in shaker at 37 °C, 180 rpm for 18 h. The bacterial culture was centrifuged twice at 6000× *g* to remove bacteria from the culture supernatant. Phage particles in the supernatant were precipitated twice with a final concentration of 30% (*w*/*v*) polyethylene glycol (PEG)/NaCl. The precipitate vector was isolated by centrifugation at 10,000× *g* for 30 min. Finally, a pellet of phage-based vector was dissolved in 2 mL of PBS, and subsequently filtered through a 0.45-µm PVDF membrane filter [19]. The phage particles were quantitated using a bacterial titration assay as described in Hajitou A., et al. [19]. Furthermore, genome copies of the phage vector were quantitated by qPCR with the modified protocol of AAV ITR titration from Aurnhammer C, et al. [20]. Briefly, phage stock was diluted in PBS (1:5000). Five microliters of the diluted phage were used as template. qPCR primers that specifically bind to ITR regions on the phage vector genome were used for detection (Table S1). The qPCR cycling conditions were: 50 °C for 2 min, 98 °C for 3 min, (98 °C for 15 s, 60 °C for 30 s) (35 cycles), followed by dissociation curve analysis. The titers from the bacterial and qPCR titration assays were expressed as transduction units per μL (TU/μL) and genome copies per μL, respectively.

### 2.11. Transduction of Primary HACs with Phage-Lucia

HACs cells were trypsinized, counted by a haemocytometer, then seeded into 96-well plates and grown for 24 h to reach 60–70% confluence. DMEM without FBS was used as the transduction medium for all cell types except Leibovitz’s L-15 without FBS for SW982. Phage-based vector stock solution was diluted in the transduction medium, and gently mixed by inversion for 5 min. Next, the cells were transduced with different amounts of phage particles carrying a secreted luciferase (*Lucia*) transgene named M13.Phage-*Lucia* for non-targeted particles and CAP.Phage-*Lucia* for targeted phages at 500,000 TU/cell. During transduction, cells were maintained in a humidified atmosphere at 37 °C and 5% CO_2_ for 24 h, then the transduction medium was replaced with 150 µL of growing medium supplemented with 10% FBS in each well, and the transduced cells were left to grow. The *Lucia* luciferase reporter protein was secreted out to the cultured medium. To monitor *Lucia* luciferase gene expression, the culture medium was collected daily from days 3 to 7 post transduction and the secreted luciferase activity was quantified using the QUANTI-Luc™ detection kit (InvivoGen, Toulouse, France) according to the manufacturer’s protocol. In brief, 25 µL of culture medium was added to an equal volume of QUANTI-Luc substrate in a luciferase-measuring 96 well plate. The plate was incubated at room temperature in the dark for 5 min. Subsequently, the luciferase activity was measured with the GloMax^®^ Navigator Microplate Luminometer (Promega, Chilworth Southampton, Hampshire, UK).

### 2.12. Statistical Analysis

All data are presented as mean ± SEM (standard error of the mean). For statistical analysis, we used the independent *t*-test, the one-way ANOVA, and the Mann–Whitney Test. Statistical significance was expressed as *p* values of * < 0.05, ** < 0.01 and *** < 0.001. All statistical analyzes were developed using SPSS software.

## 3. Results

### 3.1. CAP Peptide Selectively Binds to Primary Human Chondrocytes in a Dose-Dependent Manner

**Figure 1:**
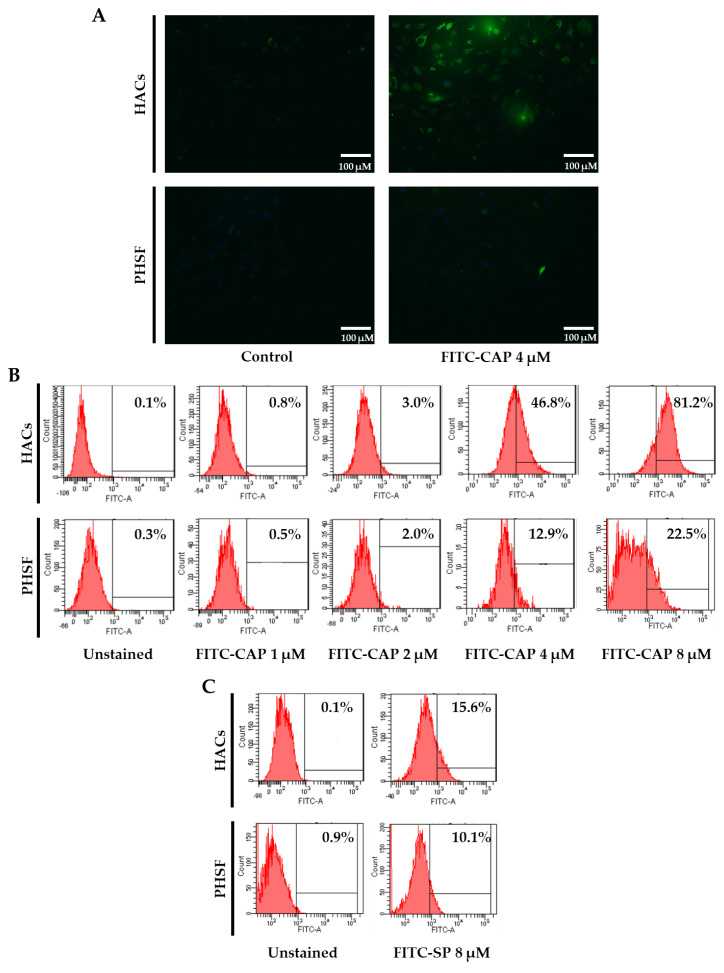

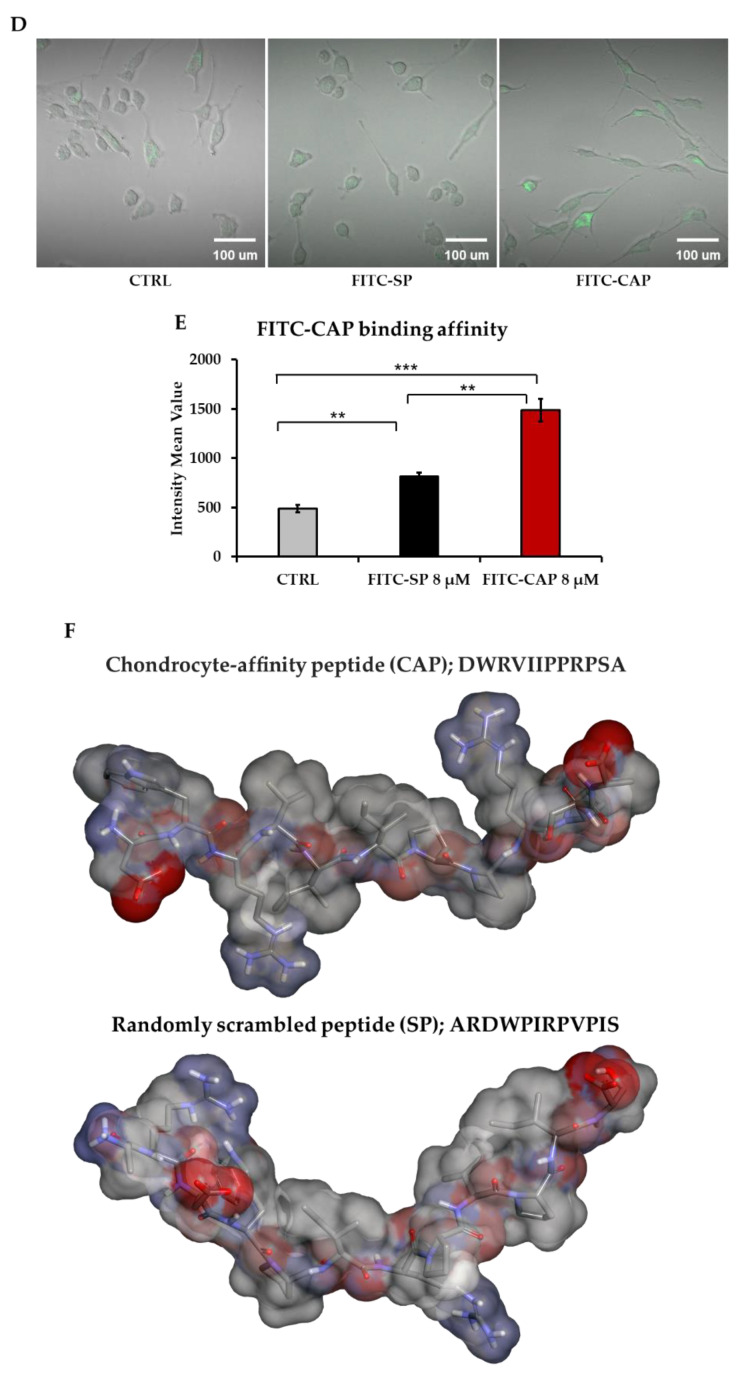
(**A**) Fluorescence microscopy imaging of FITC-positive cells. Primary HACs and PHSFs were incubated with synthetic FITC-CAP or PBS (control). (**B**) Flow cytometric analysis of FITC-positive cells was performed. HACs and PHSFs were incubated with varying concentrations of FITC-CAP (1–8 μM). (**C**) Treatment of HACs and PHSFs with 8 μM of scrambled FITC-SP. The cells were trypsinized and analyzed by flow cytometry. (**D**) Confocal fluorescence microscopic analysis of HACs. Cell was incubated with 8 μM of FITC-CAP or FITC-SP and analyzed by a confocal microscope. (**E**) The mean intensity value of the confocal microscope data is shown. Results are presented as mean ± SEM. ** represents *p* < 0.01, and *** represents *p <* 0.001, *n* = 3. (**F**) Computer generating conformation of CAP and SP. The colors represent the evolution of the donor-acceptor charge property of amino acid sequences as illustrated in blue-to-red color for donor and acceptor units.

To evaluate the selectivity of the CAP peptide, HACs and primary human synovial fibroblasts (PHSFs) were used as cellular models. HACs were isolated from the knee joint of a patient, age 11. Then, 4 μM FITC-conjugated synthesized CAP (FITC-CAP) were added to both cell cultures and incubated for 4.5 h. The binding affinity of FITC-CAP was observed under fluorescence microscope. The signal of FITC-CAP was found in the cytoplasm of HACs but barely detected in PHSFs (Figure 1A). The affinity of FITC-CAP was also quantified by flow cytometric analysis. Different concentrations of FITC-CAP (1 to 8 µM) were incubated with HACs and PHSFs for 4.5 h and subjected to flow cytometry. As shown in Figure 1B, FITC-CAP has affinity to HACs in a dose-dependent manner. The highest rate of FITC-positive cells (81.2%) was achieved when incubating with 8 µM of FITC-CAP. However, only 22.5% of FITC-positive cells were detected following treatment of PHSFs with the highest dose, 8 µM of FITC-CAP.

To demonstrate that the binding affinity of CAP are selective and dependent on the primary structure of CAP, a scrambled version of CAP (SP; ARDWPIRPVPIS) was synthesized and subsequently conjugated with FITC (FITC-SP). Next, 8 µM of FITC-SP were incubated with either HACs or PHSFs, and FITC-positive cells were quantified with flow cytometry. The results showed that only 15.6% and 10.1% of positive cells were detected upon treatment of HACs and PHSFs cells, respectively (Figure 1C). Moreover, the binding affinity of FITC-CAP was confirmed by confocal microscopy, as only the FITC-CAP signal was observed in the HACs cytoplasm (Figure 1D). We also found that HACs treated with 8 µM of FITC-CAP had two- and three-times higher mean intensity value than cells receiving FITC-SP and untreated controls, respectively (Figure 1E). These data demonstrated the specificity of CAP to HACs, which is dependent on amino acid sequence of the peptide.

According to these results, the molecular conformation of both peptide fragments was modeled (Figure 1F). The obtained conformation of these peptides may confirm the following: (i) that the CAP ligand can specifically bind to the target receptors through the unique conformation based on the amino acid alignment; (ii) that the high-content of hydrophobic amino acids (gray color) on the SP ligand may cause the low binding affinity of the ligand and affect the ability to anchor on the target receptor.

### 3.2. Pathological Condition of Primary HACs Affects the Affinity of the CAP Ligand

**Figure 2:**
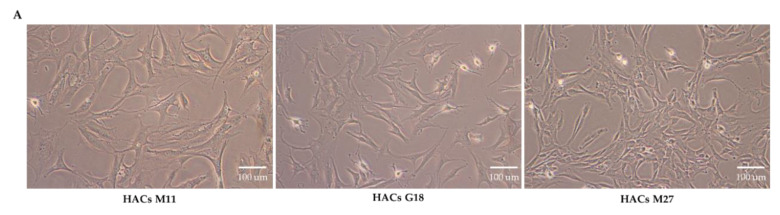

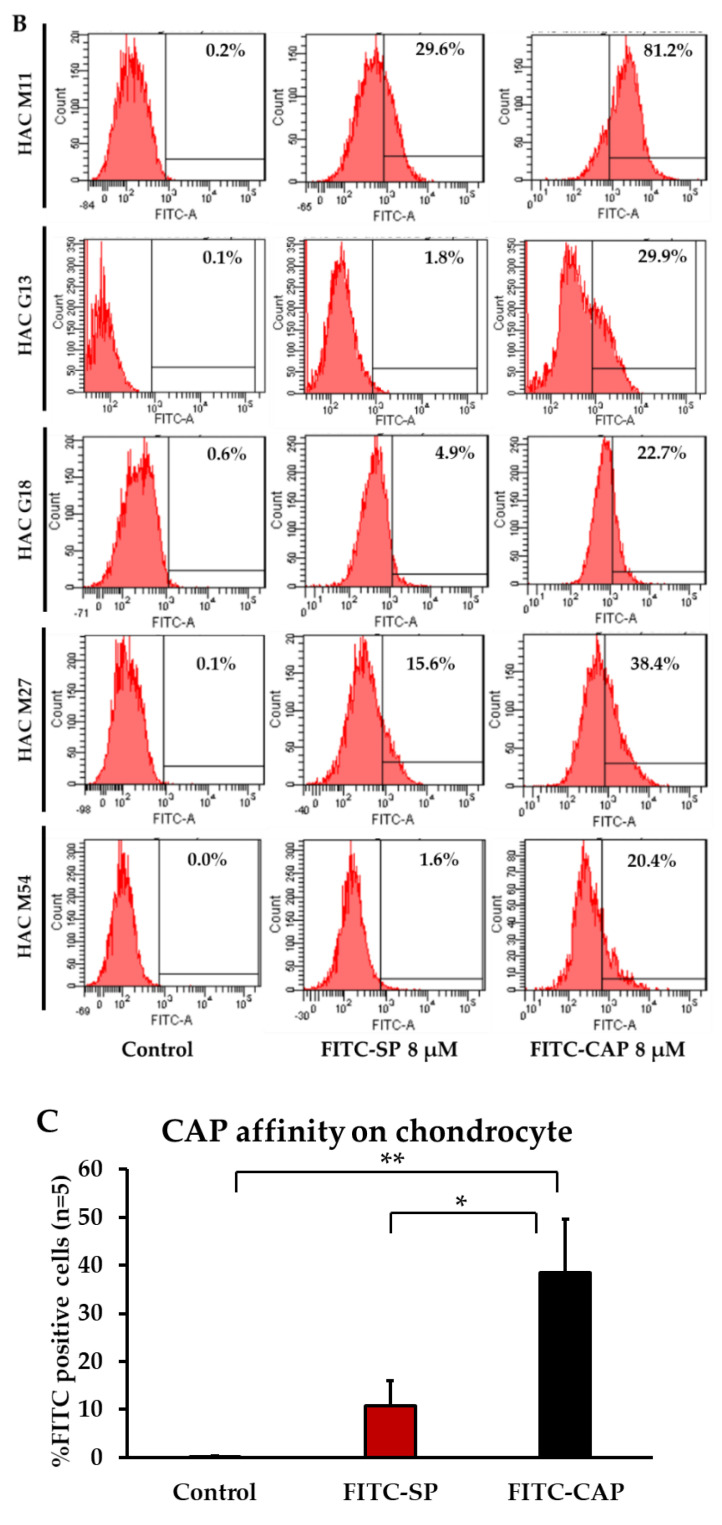

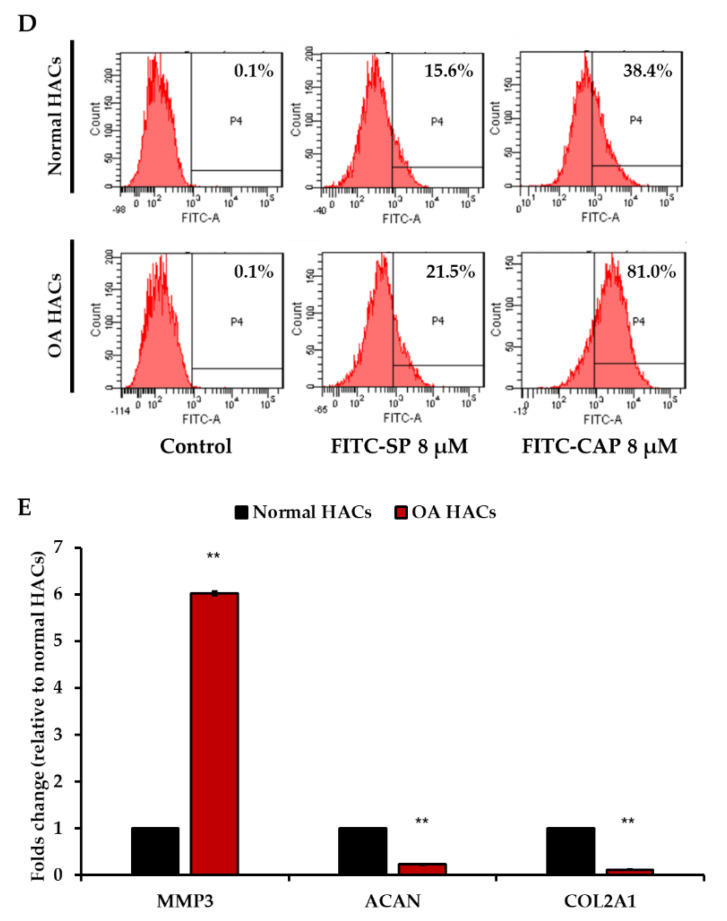
(**A**) Morphology of HACs observed under a phase contrast microscope. (**B**) Flow cytometric analysis of FITC-positive cells. Primary HACs from donors of various ages, from 11 to 54 years old, were incubated with FITC-CAP or FITC-SP, and binding affinity was analyzed by flow cytometry. (**C**) Percentage of FITC-CAP- and FITC-SP-positive HACs. (**D**) Binding affinity of FITC-CAP and FITC-SP on normal HACs and OA HACs. (**E**) Expression of OA gene markers in HACs isolated from non-pathologic and osteoarthritic lesions. Results are shown as mean ± SEM. * *p <* 0.05 and ** *p <* 0.01. Experiments were repeated 5 times (*n* = 5).

Primary HACs were isolated from donors of various ages, as shown in Supplementary Table S2, who were admitted to the hospital for total knee removal. There were no signs of abnormality in articular cartilage from the knees of the donors. We therefore evaluated whether chondrocytes from various subjects showed different affinities to the CAP peptide. We retrieved HACs from five donors aged between 11 and 54 years old, and the cells were cultured up to four passages. As illustrated in Figure 2A, chondrocytes from the three groups showed similar normal cobblestone morphology. All HACs were cultured under similar conditions until they reached 80% confluence. Either FITC-CAP or FITC-SP at 8 µM were incubated with HACs for 4.5 h at 37 °C, and the cells were subsequently analyzed by flow cytometry. As shown in Figure 2B, C, all five HACs samples took up FITC-CAP to a significantly greater degree than FITC-SP and control group (HAC alone).

Next, we investigated the binding affinity of CAP on the pathologic chondrocytes. We performed experiments by using HACs from a donor aged 27 whose articular cartilage showed signs of OA, upon confirmation by the pathological state. HACs were isolated from two different areas of the same joint, the normal cartilage area (non-pathologic group) and the OA cartilage lesion area (pathologic group). The FITC-CAP or FITC-SP were incubated with both groups of HACs, and the FITC signals were measured by flow cytometric analysis. We found that normal chondrocytes bound to the control FITC-SP peptide at 15.6%, which slightly increased to 21.5% in chondrocytes isolated from the osteoarthritic zone (Figure 2D). In the FITC-CAP-treated group, 38.4% of normal chondrocytes bound to the peptide and, importantly, up to 81.0% of chondrocytes that were isolated from the osteoarthritic zone bound to the CAP ligand (Figure 2D). To verify the characteristic of OA chondrocytes, we investigated the expression of the OA gene markers in HACs isolated from OA and normal cartilage areas by RT-qPCR. The expression of the catabolic gene matrix metalloproteinase-3 (MMP3) was significantly higher in OA HACs than normal HACs, reaching an approximately six-fold increase (Figure 2E). The expression of both anabolic genes, aggrecan (ACAN) and Collagen type2A1 (COL2A1), was significantly reduced (Figure 2E). These data demonstrate that the CAP ligand has higher affinity for osteoarthritic chondrocytes than their normal counterparts.

### 3.3. Increase in CAP Binding in Interleukin-1β (IL-1β)-Induced Inflammatory Primary HACs 

**Figure 3:**
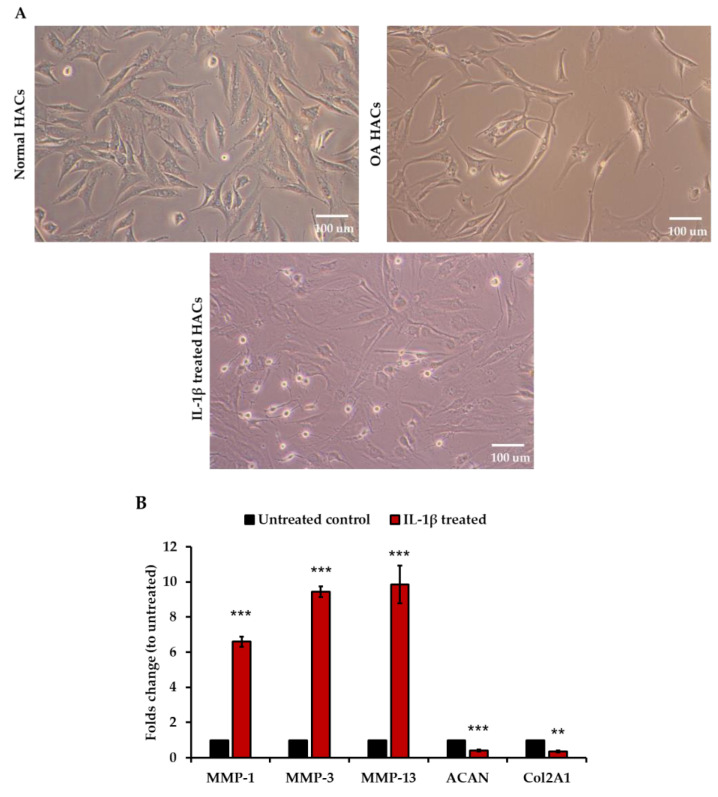

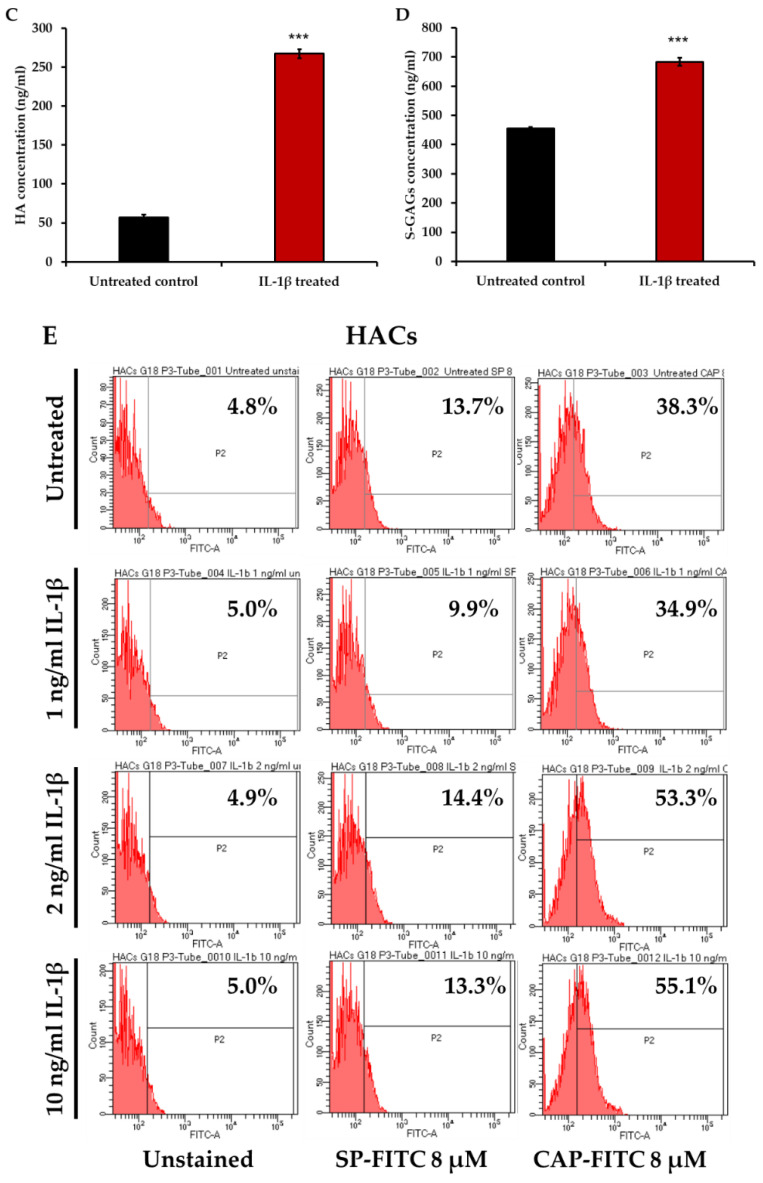
(**A**) Morphology of HACs in different conditions under a phase contrast microscope. The HACs at 80% confluence were maintained in serum-free DMEM for 24 h at 37 °C prior to treatment with 10 ng/mL IL-1β for 24 h. (**B**) Transcriptional expression of chondrocyte catabolic and anabolic genes in HACs by RT-qPCR (*n* = 3). (**C**) ELISA analysis of HA expression in cultured medium collected from HACs (*n* = 3). (**D**) ELISA analysis of S-GAGs production in the medium of HACs (*n* = 3). Results are shown as mean ± SEM. ** *p* < 0.01, and *** *p* < 0.001 compared with untreated group. (**E**) Flow cytometric analysis of HACs and IL-1β-treated HACs in various concentration at 1, 2, and 10 ng/mL. The cells were incubated with CAP-FITC or SP-FITC at 8 μM for 4.5 h at 37 °C. Next, the cells were washed and analyzed for FITC-positive cells.

In order to confirm the binding affinity of the CAP ligand into osteoarthritic HACs, we generated an osteoarthritic cellular model by treating primary HACs with IL-1β, a key cytokine involved in the pathogenesis of OA [21]. Following our previous report, we used 10 ng/mL of recombinant human IL-1β to treat normal primary HACs for 24 h. Figure 3A shows the morphology of the primary HACs prior to, and following, IL-1β treatments. The morphology of IL-1β-treated HACs is similar to normal HACS, which is cobblestone shaped. Thus, it is not possible to distinguish between IL-1β-treated HACs and the normal HACs. Therefore, we sought to assess the metabolic changes in HACs at the transcriptional and translational levels. As shown in Figure 3B, significant up-regulation of catabolic genes (*MMP-1, -3, -13*) and down-regulation of anabolic genes (*ACAN, COLII*) was observed in the IL-1β-treated HACs. In addition, the levels of secreted hyaluronan (HA) (Figure 3C) and sulfated glycosaminoglycans (s-GAGs) (Figure 3D) significantly increased in the IL-1β-treated HACs. Altogether, the IL-1β-treated HACs acquired features of osteoarthritic chondrocytes, and thus, showed the potential to be used as an in vitro model.

Next, we investigated the binding affinity of CAP by incubating the IL-1β-treated HACs with FITC-CAP or FITC-SP. Similarly to our previous experiment on primary OA HACs, we found higher CAP ligand binding in IL-1β-treated HACs, reaching 53.3% at 2 ng/mL and 55.1% at 10 ng/mL, compared to 38.3% in normal HACs (Figure 3E). Interestingly, there was no significant difference in SP ligand binding between the normal and IL-1β-treated HACs (Figure 3E). The binding affinity of CAP was also evaluated in PHSFs, as another cell type located in the joint and showing OA characteristics when induced with IL-1β [22,23]. The data showed no significant difference in CAP ligand binding between normal PHSFs, at 22.5%, and IL-1β-induced PHSFs, at 26.2%, (Supplementary Figure S1).

### 3.4. Phage-Based Vector Is a Stable Vehicle for Human Chondrocyte Gene Delivery

**Figure 4:**
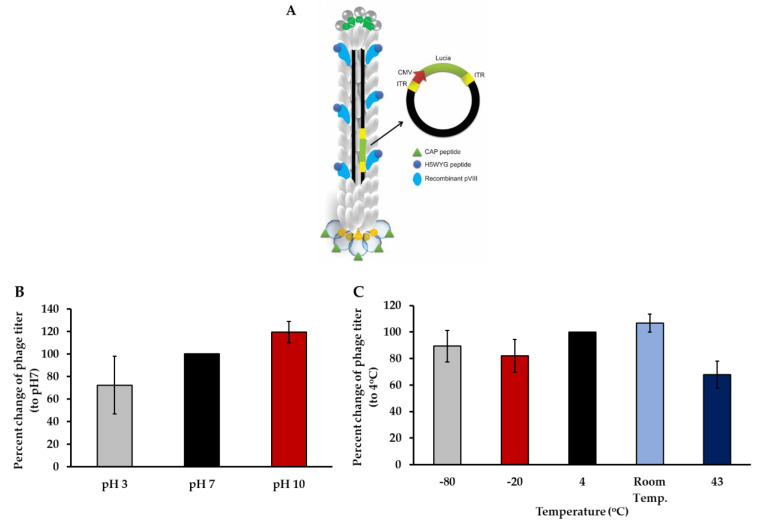

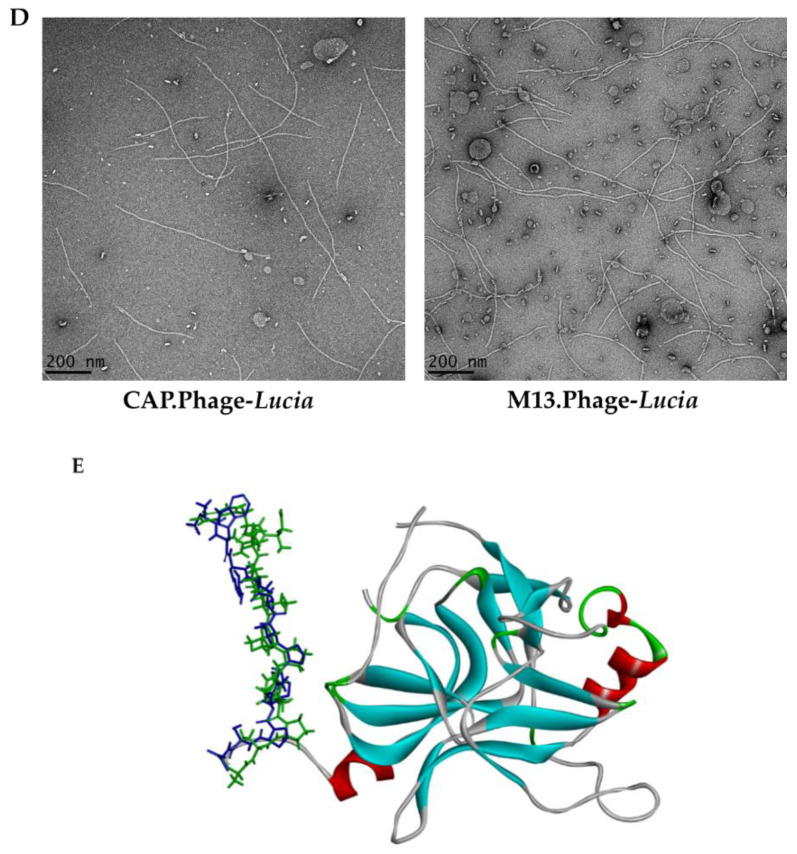
(**A**) Illustration represents targeted CAP.Phage-*Lucia* gene. (**B**) Stability of phage particles under various pH levels. Phage particles were kept in PBS at pH 3, pH 7, and pH 10 at 4 °C for 24 h (*n* = 4). (**C**) Stability of phages at different temperatures. Phage vectors were left in PBS, pH 7, at −80 °C, −20 °C, 4 °C, room temperature, and 43 °C for 24 h (*n* = 3). Next, phage titers were measured again by *E. coli* infection followed by colony counting. Results are shown as mean ± SEM. (**D**) Electron microscopic images of bacteriophage-based particles, namely M13.Phage-*Lucia* and CAP.Phage-*Lucia*, at 20,000× magnification. (**E**) Computer modelling of the pIII minor coat protein expressing CAP (green) compared with the free form of CAP (blue).

The ideal nanocarrier for nucleic acid delivery should be non-toxic, stable, and precisely targeted to the cell of choice. We therefore combined CAP with a phage-based vector and evaluated its potential to deliver transgene expression to primary HACs. We genetically engineered our M13 phage-based vector by inserting a DNA sequence encoding the CAP ligand in frame with the M13 phage pIII gene expressing minor coat proteins on the phage capsid. In addition, a DNA sequence encoding the endosomal escape peptide (H5WYG) was inserted in frame with the recombinant pVIII gene [14]. Thus, the CAP and H5WYG ligands were displayed on the pIII and pVIII phage coat proteins, respectively. Next, a CMV promoter-guided mammalian transgene cassette, flanked by AAV-2 ITRs, was inserted into the genome of the phage-based vector [19]. As shown in Figure 4A, CAP and H5WYG displayed phage-based vectors, and CAP.H5W.Phage was propagated in *E. Coli* and subsequently purified with PEG/NaCl precipitation. The wild type phage vector with H5WYG ligands but without CAP was used as a control (non-targeted phage-based vector). Titers of phage were obtained by two different titration methods using the colony-forming assay to determine the viable phages [19], and quantification of the genome copies by qPCR to evaluate the total phages [20]. The titration results showed that the phage vector displaying the CAP ligand on the pIII minor coat proteins had slightly lower titer than that of the wild type M13 H5WYG phage-based vector when measured using the bacterial titration method (Supplementary Table S3).

The stability of delivery systems is a major challenge faced by gene therapy. Lentivirus, adenovirus, and AAV are very sensitive to temperature and pH, requiring ultra-low temperature storage conditions. We therefore evaluated the stability of the phage-based vector under different pH levels and temperatures. First, 1 × 10^10^ TU of phage vector was kept in PBS solution at three different pH conditions (3, 7 and 10) for 24 h at 4 °C. Next, these phages were used to infect *E. coli* in order to re-quantitate the phage titer and evaluate the viable intact phage particles. Compared to the phage titer observed prior to treatment at pH 7, no change in titer was observed when the phage was exposed to pH 10 (Figure 4B). However, we lost 28% of the phage titer in conditions at pH 3.

Next, we investigated the effects of various temperatures on the phage titer. Phage vectors are commonly stored at 4 °C. Thus, we sought to monitor the phage titers under different temperatures ranging from −80 to 43 °C for 24 h, without any anti-freezing agent and compared to the phage titer kept at 4 °C. As shown in Figure 4C, no significant loss of the phage titers was detected at −80, −20, or 25 °C temperatures. Interestingly, a temperature of 43 °C resulted in 38% loss of the phage titer.

As illustrated in Figure 4D, we performed electron microscopy to visualize the CAP.H5WYG.Phage particle and control the M13.H5W Phage (wild type) particle. We found that the CAP.Phage particles showed an approximate diameter of 800 nm, similar to the unmodified M13.Phage particle. Altogether the data demonstrated that phage particles are stable under harsh conditions of extreme pH and temperature, and that modification of the phage capsid does not affect the particle production, integrity, or structure. Furthermore, the 3D structure of the pIII minor coat proteins was modelled to predict the CAP conformation after its display on the coat proteins (Figure 4E). In a comparison of the regions of the CAP ligand, it was found that the configuration of CAP between the free and displayed form on the pIII minor coat protein is slightly different along the N- to C-terminal amino acid chains.

### 3.5. CAP.Phage Based Vector Is a Non-Toxic and Efficiently Delivered Transgene to Primary HACs

**Figure 5:**
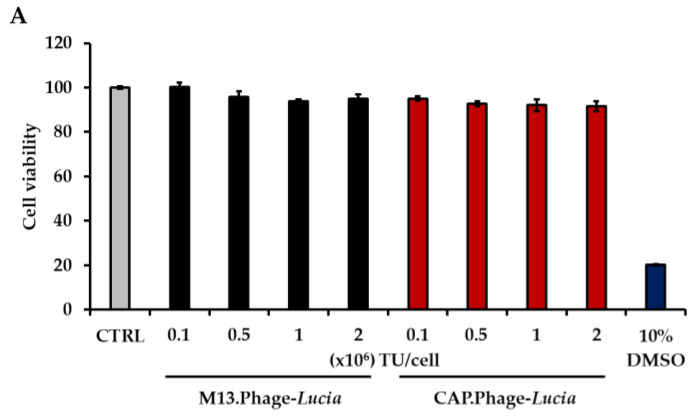

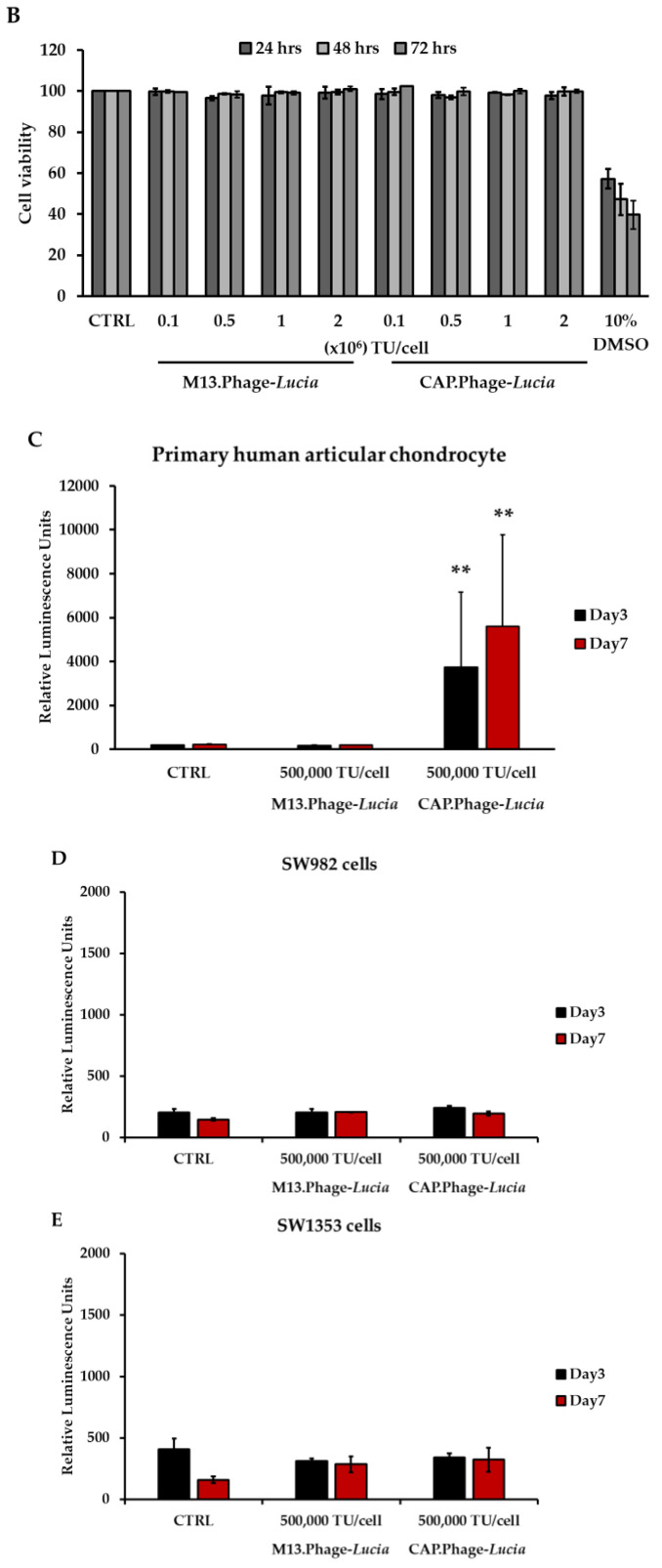
CAP.Phage cytotoxicity analysis on HACs. The cells were treated with CAP.Phage-*Lucia* and control M13.Phage-*Lucia* ranging from 0.1 × 10^6^ TU/cell to 2 × 10^6^ TU/cell for 24 h at 37 °C, then cell viability was measured using the MTT assay (*n* = 4) (**A**) and Trypan Blue Exclusion Test (*n* = 2) (**B**). Efficacy analysis of CAP.Phage-mediated gene delivery using a secreted luciferase reporter gene on HACs (*n* = 6) (**C**), SW982 (*n* = 3) (**D**) and SW1353 (*n* = 3); the Mann–Whitney Test was used for statistical analysis of cells (**E**). The cells were treated with CAP.Phage-*Lucia* or M13.Phage-*Lucia* at 0.5 × 10^6^ TU/cell for 24 h at 37 °C. Culture media were replaced with 10%FBS DMEM and incubated at 37 °C. At days 3 and 7 post-transduction, the culture media were collected and used to analyze the production of secreted luciferase reporter using the QUANTI-Luc™ detection kit (InvivoGen, France) and GloMax^®^ Navigator Microplate Luminometer (Promega, Chilworth Southampton, Hampshire, UK). Results are shown as mean ± SEM., ** *p* < 0.01 compared with M13.Phage-*Lucia*.

Cytotoxicity caused by delivery vectors is a major concern in several gene delivery platforms. Mammalian viral vectors, for example the adenoviral vector or chemically based nanocarriers, can cause cytotoxicity to targeted cells [24,25]. Our previous results showed that the phage-based vector has no cytotoxicity in both in vitro and in vivo experiments [14]. To confirm this, we evaluated the cytotoxicity of the CAP.Phage vector. Different concentrations of phage-based vectors, ranging from 0.1 to 2 × 10^6^ TU/cell, were incubated with primary HACs for 24 h. The cytotoxicity was then determined using the MTT assay and the Trypan Blue Exclusion Test. As shown in Figure 5A,B, both the M13.Phage vector and the CAP.Phage vector did not show any significant cytotoxicity when exposed to primary HACs. This result confirmed that the CAP.Phage vector is not toxic to HACs. Next, we assessed the gene delivery efficiency of the CAP.Phage vector. We used vector-expressing *Lucia* as a reporter gene. The *Lucia* gene was inserted into the mammalian transgene cassette of the CAP.Phage vector. The CAP.Phage vector carrying the *Lucia* gene (CAP.Phage-*Lucia*) at 0.5 × 10^6^ TU/cell was used to transduce HACs cells. Non-targeted phage vector (M13.Phage-*Lucia*) was used as a control. At 24 h post-phage treatment, the transduction medium was removed and replaced with regular HACs culture medium. At days 3 and 7 post-transduction, cultured media were collected, and luciferase activity was determined. As demonstrated in Figure 5C, only the CAP.Phage vector induced *Lucia* expression in HACs, both at days 3 and 7, reaching 3729 and 5594 relative luminescence units (RLU), respectively. No *Lucia* activity was observed in both untreated cells and cells receiving the non-targeted M13.Phage-*Lucia*. To further confirm the selectivity of the CAP.Phage vector for human chondrocytes, we performed a similar experiment on different cell lines, including the SW1353 and SW982 chondrosarcoma cell lines. As shown in Figure 5D,E, no *Lucia* activity was observed in both the SW1353 and SW982 cell lines when transduced with the CAP.Phage-*Lucia* or M13.Phage-*Lucia* vector at 0.5 × 10^6^ TU/cells, respectively. Taken together, these results confirm that the CAP.Phage vector efficiently targets and delivers transgenes to primary human chondrocytes.

## 4. Discussion

The chondrocyte-affinity peptide (CAP) was first identified in 2011 by phage display technology, and CAP-conjugated polyethylenimine (PEI) was found to efficiently deliver DNA plasmid to chondrocytes [8]. However, PEI is undegradable and highly toxic [26,27]. Herein, we show that the M13 bacteriophage is a good candidate to replace the toxic PEI polymer as a carrier for therapeutic nucleic acid. The CAP ligand can be promptly displayed on pIII minor coat proteins, avoiding chemical conjugation. Moreover, the transgene is wrapped inside the phage particle, protecting the transgene from DNases and the harsh extracellular environment. In this study, we showed that no cytotoxicity was observed in phage-vector-treated HACs, even though HAC cells were incubated with very high doses of the vector. An in vivo toxicity study of the phage vector was previously performed in mouse models, where histopathological and serological analyses in different organs showed no toxicity in animals receiving a therapeutic dose of phage vector at 5 × 10^10^ TU per animal [13]. In humans, phages have been used to fight against antibiotic-resistant infections and showed promising outcomes [28,29]. In 2019, a patient with cystic fibrosis with a disseminated *Mycobacterium abscessus* infection was intravenously treated with phages and showed an excellent resolution without any side effects [30]. These studies confirm the safety of phage treatment in humans.

In a previous study, the affinity of the CAP ligand to human chondrocytes was investigated with the sample from only one single male donor with osteoarthritic condition, and the passages of cell culture were unknown [8]. In this study, we confirmed these properties of the CAP ligand from five different donors at different ages and genders. In addition, we limited the passage numbers of HAC culture up to four passages because chondrocytes appear to lose their phenotype beyond this number of passages. Multiple studies showed that aging chondrocytes exhibit senescence phenotypes, which alter the expression of genes involved in cell proliferation [31], the synthesis of matrix biomolecules [32], and the expression of cell surface molecules [33]. Interestingly, we also found that chondrocytes from the youngest age subject show a similar binding affinity to the OA group. To confirm whether the binding affinity of CAP ligand correlates with the age of chondrocytes, more sample numbers of chondrocytes are required. Furthermore, elucidation of the unknown receptor will be very helpful to understand the interaction between the CAP ligand and chondrocyte.

Interestingly, HACs isolated from the same joint, with or without OA characteristics, show different binding affinities to CAP ligands. HACs isolated from the OA zone can bind to the CAP ligand significantly more than HACs from the normal zone. Due to the limitation of the natural OA chondrocytes, we confirmed this event by using the IL-1β-induced inflammatory OA model. The IL-1β treatment of chondrocytes results in OA-like pathogenesis and plays a pivotal role in the metabolic changes of chondrocytes [34]. The elevation of IL-1β in synovial fluids and joint tissues is related to inflammation and cartilage destruction [35]. Similar to the natural HACs with OA condition, the IL-1β-treated HACs can bind to the CAP ligand significantly more than the untreated HACs. Markedly, no such effect was observed when we conducted a similar experiment on PHSFs. The metabolic changes in OA chondrocytes may lead to better accessibility of the CAP, which could be associated with cell surface receptor expression, as well as reduced extracellular matrix (ECM) production and deposition [36,37]. As ECM is a major barrier to successful gene therapy, we previously showed that modulation of the ECM by enzymatic digestion enhances targeted gene delivery by phage [38]. In the same manner, OA cartilage has less ECM production and deposition. This allows therapeutic molecules or delivery vectors to access the pathogenic site more easily than the normal site. Taken together, the CAP ligand has high affinity towards OA chondrocytes and serves as a promising ligand for chondrocyte-targeted phage-based delivery. Due to the limitation in recruiting the OA chondrocyte samples, further investigation to confirm the affinity of CAP to OA chondrocytes with higher sample numbers shall be conducted.

Phage-based vectors have potential advantages over other common eukaryotic viral vectors for gene therapy purposes, in terms of production, modification, and stability [19]. Production of eukaryotic virus requires an expensive mammalian cell culture system. The cost is substantially higher than bacteriophage production cost, which can be produced using a cost-effective bacterial culture system [39,40,41,42]. In addition, the bacteriophage production and isolation can be conducted within two days whereas the eukaryotic viruses require at least one week [19,43,44].

Given that bacteriophages do not naturally infect mammalian cells, their capsids can be modified to display a ligand that specifically binds to a receptor displayed on the target cell. Unlike other mammalian viruses, the modification of capsids does not alter the overall structure and titer of the phage vector. Based on previously reported phage based-vectors [19], we generated a version of phage for proof-of-concept gene delivery to osteoarthritic chondrocytes. We genetically modified the pIII minor coat proteins of the phage to display the CAP as a targeting ligand for OA HACs. Besides ECM, endosome-lysosome degradation is another major barrier for gene delivery by phages since phage-based vectors are not equipped with endosomal escape molecules, as is the case for other mammalian viruses. Thus, we engineered a phage-based vector with a histidylated fusogenic peptide, H5WYG, derived from the HA-2 subunit of the influenza virus hemagglutinin [45]. The peptide facilitates the endosomal escape of phage particles via permeabilization of the endosome membranes. Our previous study demonstrated that displaying the H5WYG ligand on the recombinant pVIII major coat proteins of filamentous phage facilitates endosomal escape and enhances transgene expression [14]. Finally, our OA chondrocyte-targeted phage-based particle is equipped with CAP and H5WYG ligands to target and enhance escape from endosomes, respectively. The phage DNA construct also contains a mammalian transgene cassette flanked by the ITRs element from AAV2. We found that modification of the phage capsid does not affect the structure and viability of the phage particles. There is no difference in size and shape of phage under electron micrograph and no significant alteration of the phage titer between wild type phages and the CAP.Phage. Many mammalian viral vectors are sensitive to harsh conditions including pH and temperature. They require ultra-low temperatures. Moreover, freeze and thaw cycles vastly affect the viability of the vectors. Unlike mammalian viruses, our phage-based particle is stable under various pH and temperatures. Storage of phage-based vectors under 4 °C only causes a slight reduction in the phage titer at approximately one log decrease after a year (Figure S2).

We used a secreted luciferase (*Lucia)* as a reporter gene to evaluate the efficacy of CAP.Phage and showed that CAP.Phage-*Lucia* can target and deliver transgenes to HACs. Interestingly, the reading values of *Lucia* activity in CAP.Phage-*Lucia*-transduced HACs showed high variation between experiments. We speculate that the extracellular matrix produced by HACs may hinder accessibility of the phage particles to HAC cells. A drawback of many nucleic delivering particles is cytotoxicity, especially in chemically based delivery methods. Several attempts have been made to lower the toxicity of vectors while compromising gene transfer efficiency [46,47,48]. In terms of specificity to target cells, we proved that the CAP.Phage vector only delivered transgenes to HACs. We conducted similar experiments in the chondrosarcomas (SW1353) and synovial fibroblasts (SW982) cell lines, where we found that those cells are not transducable by the CAP.Phage. In future studies, replacement of the *Lucia* gene with a therapeutic gene will be undertaken to test the efficacy of the CAP.Phage vector. Transforming growth factor-beta (*TGF-β*) gene is a good candidate because it has been studied in a clinical trial of ex vivo OA gene therapy and showed positive outcomes [49,50,51].

Based on the molecular modeling findings, the CAP structure was slightly changed when displayed on the pIII minor coat proteins; nevertheless, the selectivity and transduction ability of the CAP.Phage was still preserved. We inserted the CAP encoding DNA sequence into the N-terminal of pIII minor coat proteins with GGGGS (Gly-Gly-Gly-Gly-Ser) DNA linker. The linker provides prodigious flexibility to the displayed peptide and has been successfully used in many studies [52,53,54]. This linker may supply a flexibility to the CAP peptide and facilitates the binding of the peptide to the cell receptors on chondrocytes. Although CAP is an excellent ligand for chondrocyte targeting, its binding partner is yet to be identified. As we mentioned previously, further investigations of CAP.Phage in terms of molecular interaction with cell receptors and functionality are required in the future.

## 5. Conclusions

In summary, we designed and characterized a targeted gene delivery platform for human chondrocytes, using a combination of phage-based technology and chondrocyte-affinity peptide (CAP). For the first time, we showed that phage-based particles can deliver transgene to the HAC. Significantly, the phage-based particle showed positive outcomes as a targeted delivery tool for HACs in the aspects of safety, selectivity, and transduction efficiency. Future work should aim at evaluating the gene therapy efficacy of the CAP.H5WYG.Phage vector. This novel targeted delivery platform has potential in terms of delivery to chondrocytes and in the treatment of cartilage-related diseases.

## Data Availability

Data is contained within the article.

## Supplementary Materials

The following are available online at https://www.mdpi.com/article/10.3390/v13122343/s1, Figure S1: Flow cytometric analysis of FITC-positive PHSF cells, Figure S2: Phage stability was estimated using bacterial infection method, Table S1: Primer sequences used for real-time PCR experiments, Table S2: HACs donors’ information, Table S3: The titration result of CAP.Phage and M13.Phage.
