# Supplementary material for: Targeting Human Osteoarthritic Chondrocytes with Ligand Directed Bacteriophage-Based Particles
Source: Viruses. 2021 Nov 23;13(12):2343. doi: 10.3390/v13122343 (PMC8706358; doi:10.3390/v13122343)
Supplement: Supplementary file 1 [file viruses-13-02343-s001.zip › viruses-1450758-supplementary.pdf]

Supplementary Materials

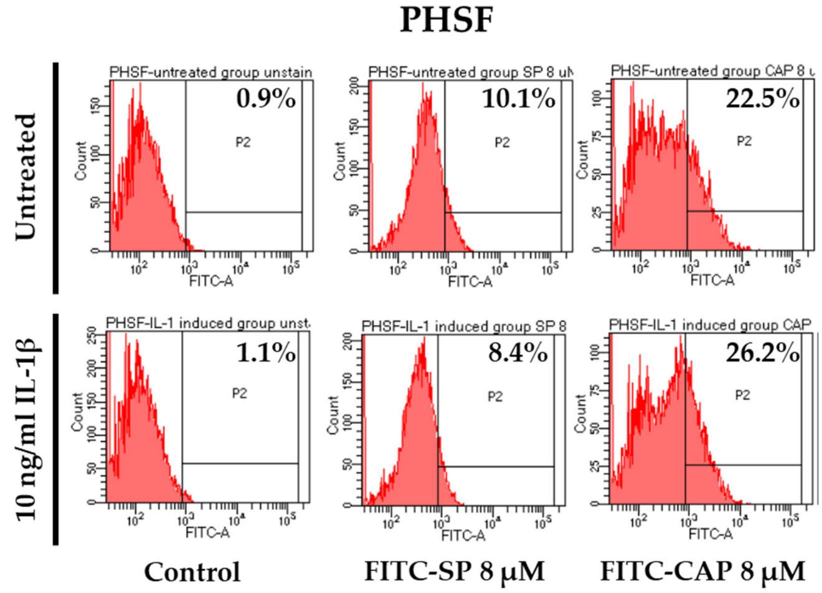

Figure S1. Flow cytometric analysis of FITC positive PHSF cells. IL-1β treated PHSF were incubated with CAP-FITC or SP-FITC at 8μM for 4.5 hours at 37°C. The cells were washed and FITC positive cells were analyzed by flow cytometry.

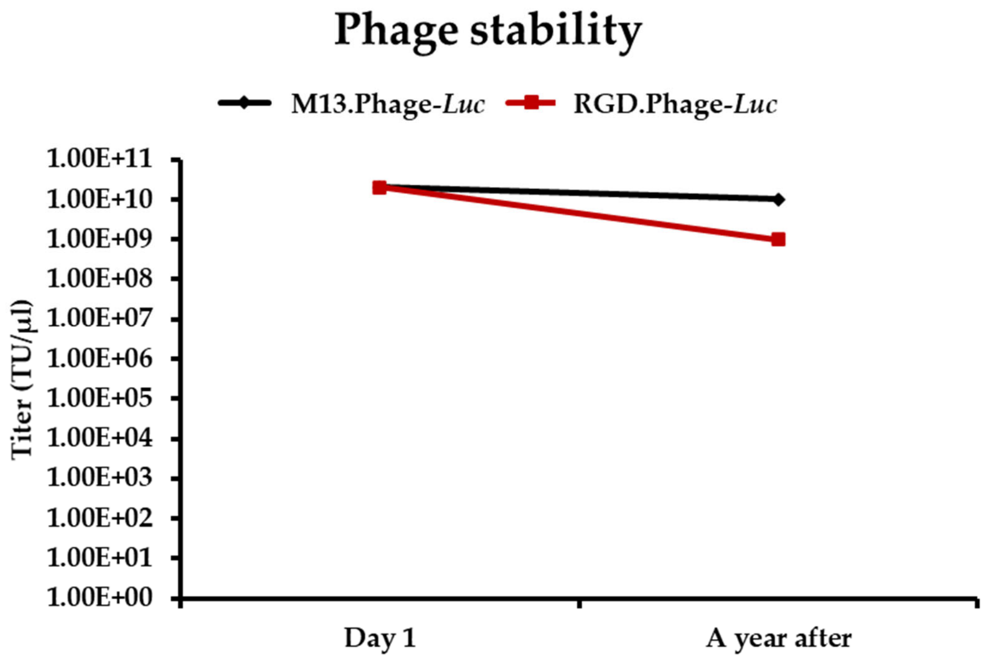

Figure S2. Phage stability was estimated using bacterial infection method. RGD.Phage-*Luc* and M13.Phage-*Luc* titers were quantified and the phage solutions were kept at 4°C for approximately 1 year. The phage solutions were then titrated again with the same bacterial infection method.

Table S1. Primer sequences used for cloning and real-time qPCR experiments.

Gene (human)	Primers (5′→3′)
CAP-pIII	Forward: *CGCCCGAGCGCGGGAGGAGGAAGCGAAACTGTTGAAAGTTGTTT*
	Reverse: *CGGCGGAATAATCACGCGCCAATCAGCGGAGTGAGAATAGAAAGG*
ITR	Forward: *GGAACCCCTAGTGATGGAGTT*
	Reverse: *CGGCCTCAGTGAGCGA*
MMP-1	Forward: *ATGAAGCAGCCCAGATGTGGAG*
	Reverse: *TGGTCCACATCTGCTCTTGGCA*
MMP-3	Forward: *AGCAAGGACCTCGTTTTCATT*
	Reverse: *GTCAATCCCTGGAAAGTCTTCA*
MMP-13	Forward: *TTGTTGCTGCGCATGAGTTCG*
	Reverse: *GGGTGCTCATATGCAGCATCA*
COL2A1	Forward: *AATTCCTGGAGCCAAAGGAT*
	Reverse: *AGGACCAGTTGCACCTTGAG*
ACAN	Forward: *AGGCAGCGTGATCCTTACC*
	Reverse: *GGCCTCTCCAGTCTCATTCTC*
GAPDH	Forward: *CCCCTTCATTGACCTCAACTAC*
	Reverse: *GATGACAAGCTTCCCGTTCTC*

Table S2. HACs donors' information

Code name	Gender	Age	Indication
HACs M11	Male	11	Osteosarcoma
HACs G13	Female	13	Osteosarcoma
HACs G18	Female	18	Osteosarcoma
HACs M27	Male	27	Chondrosarcoma at pelvis Lt.
HACs M54	Male	54	No information

Table S3. The titration result of CAP.Phage and M13.Phage

Method	Name	Titer
qPCR (genome copies/ µl)	M13.Phage-*Luc*	$2.58x10^{11}$
	CAP.Phage-*Luc*	$2.61x10^{11}$
Bacterial titration (TU/ µl)	M13.Phage-*Luc*	$3x10^{11}$
	CAP.Phage-*Luc*	$1x10^{11}$
